# APOE4 Alters Early Transcriptional Programs and Inflammatory Signaling in Human Induced Pluripotent Stem Cells

**DOI:** 10.3390/ijms27104325

**Published:** 2026-05-12

**Authors:** Wiebke Schulten, Nele Johanne Czaniera, Mehran Fazel, Barbara Kaltschmidt, Christian Kaltschmidt

**Affiliations:** 1 Department of Cell Biology, University of Bielefeld, 33615 Bielefeld, Germany; wiebke.schulten@uni-bielefeld.de (W.S.); nele.czaniera@uni-bielefeld.de (N.J.C.); mehran.fazel@uni-bielefeld.de (M.F.); c.kaltschmidt@uni-bielefeld.de (C.K.); 2 Forschungsverbund BioMedizin Bielefeld, Ostwestfalen-Lippe (OWL) (FBMB E.V.), 33617 Bielefeld, Germany; 3 Molecular Neurobiology, University of Bielefeld, 33615 Bielefeld, Germany

**Keywords:** Alzheimer’s disease, iPSCs, APOE4, RNA sequencing, neurodevelopment, differentiation, EGR, TNFR, metallothionein

## Abstract

The *APOE4* allele represents the strongest genetic risk factor for late-onset Alzheimer’s disease (AD), yet its influence on early cellular programs remains poorly understood. In this study, we investigated transcriptional differences between human induced pluripotent stem cells (iPSCs) carrying the *APOE3* or *APOE4* genotype. RNA sequencing revealed pronounced genotype-dependent transcriptional changes, with enrichment of genes associated with neural development and metallothioneins in *APOE4* cells, while genes related to extracellular matrix organization and cell adhesion were downregulated. Protein–protein interaction network analysis confirmed the presence of clusters linked to neurodevelopmental processes and cellular stress responses in *APOE4* cells. Increased expression and nuclear localization of the early neural marker SOX1 further suggest a shift towards early neural lineage commitment in *APOE4* cells. In addition, altered expression of early growth response (*EGR*) transcription factors and reduced TNFR2 protein levels indicated genotype-specific differences in stress and inflammatory signaling pathways. Together, these findings suggest that *APOE* genotype-dependent alterations in transcriptional regulation, stress responses, and inflammatory signaling may already emerge in pluripotent cells and potentially influence early differentiation programs.

## 1. Introduction

Embryonic stem cells (ESCs), derived from the inner cell mass of pre-implantation blastocysts, represent a pluripotent cell population that is extensively employed in studies of early human development, high-throughput pharmacological screening, regenerative medicine, and disease modeling [1]. Despite their utility, the use of ESCs remains ethically contentious and is subject to stringent regulatory frameworks in several jurisdictions [1]. Induced pluripotent stem cells (iPSCs) were developed as a technical accessible and ethically less controversial alternative to embryonic stem cells, generated by the enforced expression of defined transcription factors in somatic cells to reprogram them into a pluripotent state comparable to that of ESCs [2,3]. An alternative approach, somatic cell nuclear transfer (SCNT), achieves reprogramming through nuclear transfer into an enucleated oocyte, but remains technically challenging and ethically debated [4,5,6]. Genome editing approaches permit the introduction of disease-associated alleles, enabling the recapitulation of pathological genotypes in both the pluripotent and differentiated cellular contexts [7]. For instance, iPSC-derived models harboring the Alzheimer’s disease-associated risk allele *APOE4* (Apolipoprotein E4) facilitate mechanistic investigations of genotype-phenotype correlations in relevant neuronal populations [8,9].

Alzheimer’s disease (AD) was first introduced in a 1906 lecture by the German psychiatrist and neuropathologist Alois Alzheimer, based on his observations of a patient with progressive memory loss and cognitive decline [10]. In post-mortem brain tissue, he identified characteristic neuropathological changes, including extracellular “senile plaques” (now known to be largely composed of aggregated amyloid-β peptides) and intraneuronal fibrillary alterations later termed neurofibrillary tangles (primarily composed of hyperphosphorylated tau protein). These lesions remain central histopathological hallmarks of AD [11,12,13]. Furthermore, Konrad Beyreuther and colleagues determined the amino-acid sequence of the amyloid-β (Aβ) peptide [14] and subsequently cloned the cDNA encoding its precursor, the amyloid precursor protein (APP) [15]. Neurodegeneration in AD is associated with progressive neuronal and synaptic loss, leading to marked brain atrophy. The shrinking is especially pronounced in the cerebral cortex and limbic structures, including the hippocampus and entorhinal cortex, which are critical for learning and memory. Macroscopically, this atrophy manifests as widened sulci and enlarged ventricles. While grey matter (containing neuronal cell bodies) is prominently affected, white matter changes and disruptions of neural connectivity also occur and contribute to clinical symptoms [16,17,18].

Early-onset AD, which can occur as early as the fourth decade of life, was linked to autosomal-dominant inheritance through pathogenic mutations in *APP* as well as in components of the amyloidogenic proteolytic processing pathway, most notably *PSEN1* and *PSEN2* (presenilin 1 and 2), which encode catalytic subunits of the γ-secretase complex [19,20,21,22,23]. Furthermore, late-onset (sporadic) AD (LOAD), typically manifesting after age 65–70, has a substantial genetic component, with heritability estimates of up to 80% [24]. The strongest and most consistently replicated genetic risk factor for LOAD is the *APOE4* allele [20,25,26]. The presence of the *APOE4* allele increases AD risk, with homozygous carriers exhibiting a higher risk and earlier age at onset than heterozygous carriers. A recent study in individuals, homozygous for *APOE4*, reported that by age 65, almost all participants showed abnormal cerebrospinal fluid Aβ biomarkers, consistent with near-complete penetrance of amyloid pathology in this genotype [27]. In this context, CRISPR/Cas9 genome editing has been used to generate AD-relevant cellular models by introducing the *APOE4* allele into human iPSCs previously harboring *APOE3*. This isogenic allele switch exerted a pronounced effect on the transcriptomes of iPSC-derived neurons and glial populations, significantly altering the expression of hundreds of genes. Differentially expressed pathways were particularly enriched for synaptic structure and function in neurons, lipid and cholesterol metabolism in astrocytes, and innate immune responses in microglia-like cells [8]. More recently, profound transcriptome dysregulation has been reported in iPSCs, presumably driven by reduced expression of the Repressor Element-1 Silencing Transcription Factor (REST), accompanied by aberrant upregulation of neuronal lineage (proneuronal) genes in otherwise pluripotent cells [28]. This might lead to accelerated neural differentiation and diminished self-renewal of progenitor cells, accompanied by increased electrical excitability that persisted even after 3 months of differentiation.

Recently, we detected markedly elevated apoptosis rates in homozygous *APOE4* iPSCs (28%) compared with homozygous *APOE3* iPSCs (10%) [29]. Moreover, apoptosis was strongly reduced 60 min after Tumor Necrosis Factor alpha (TNF-α) treatment in *APOE3* cells (3%) but not in *APOE4* cells (remaining at 17%). Consistently, TNF-α failed to stimulate the cell-protective transcription factor Nuclear Factor kappa-light-chain-enhancer of activated B cells (NF-κB) activation in *APOE4* cells. Likewise, TNF-α–mediated induction of selected NF-κB target genes such as Interleukin-1 beta (*IL-1B)*, C-X-C Motif Chemokine Ligand 10 (*CXCL10)*, C-C motif chemokine ligand 2 (*CCL2*), and Cyclooxygenase-2 (*COX2)* was blunted in *APOE4* cells.

Here, we analyzed gene expression in *APOE4* and *APOE3* iPSCs. We observed a marked upregulation of pro-neuronal regulatory genes, metallothionein (MT) genes and a simultaneous down-regulation of *EGR1–EGR4* and typical extracellular matrix and adhesion genes in *APOE4* cells compared to *APOE3* cells.

## 2. Results

### 2.1. APOE Genotype Drives Global Transcriptional Differences in iPSCs

**Figure 1:**
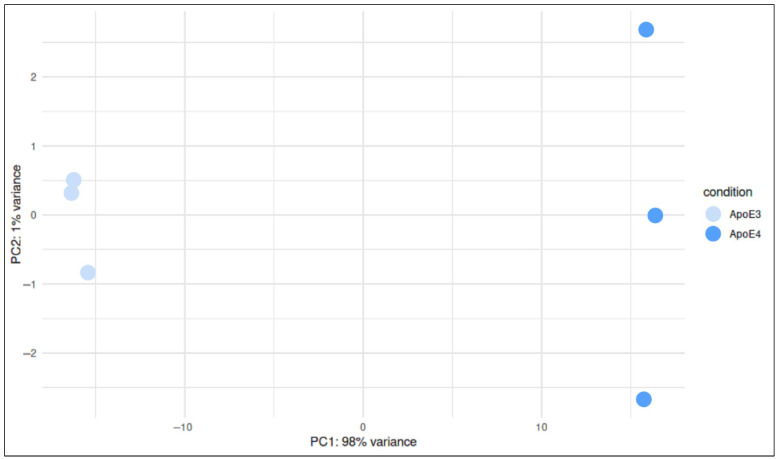
Principal component analysis (PCA) of *APOE3* and *APOE4* iPSC RNA samples. The plot shows the distribution of samples along principal component 1 (PC1) and principal component 2 (PC2), which explain 98% and 1% of the total variance, respectively. Each point represents a technical replicate (*n* = 3), colored according to the genotype. PC1 accounts for the majority of variation between both genotypes *APOE3* and *APOE4*.

RNA sequencing was performed to investigate transcriptional differences between *APOE3* and *APOE4* iPSCs. To evaluate global transcriptional variability across samples, we performed principal component analysis (PCA) (Figure 1). PC1 and PC2 explained 98% and 1% of the total variance, respectively. Samples clustered according to *APOE* genotype, indicating that this factor represents a major source of transcriptional variation.

**Figure 2:**
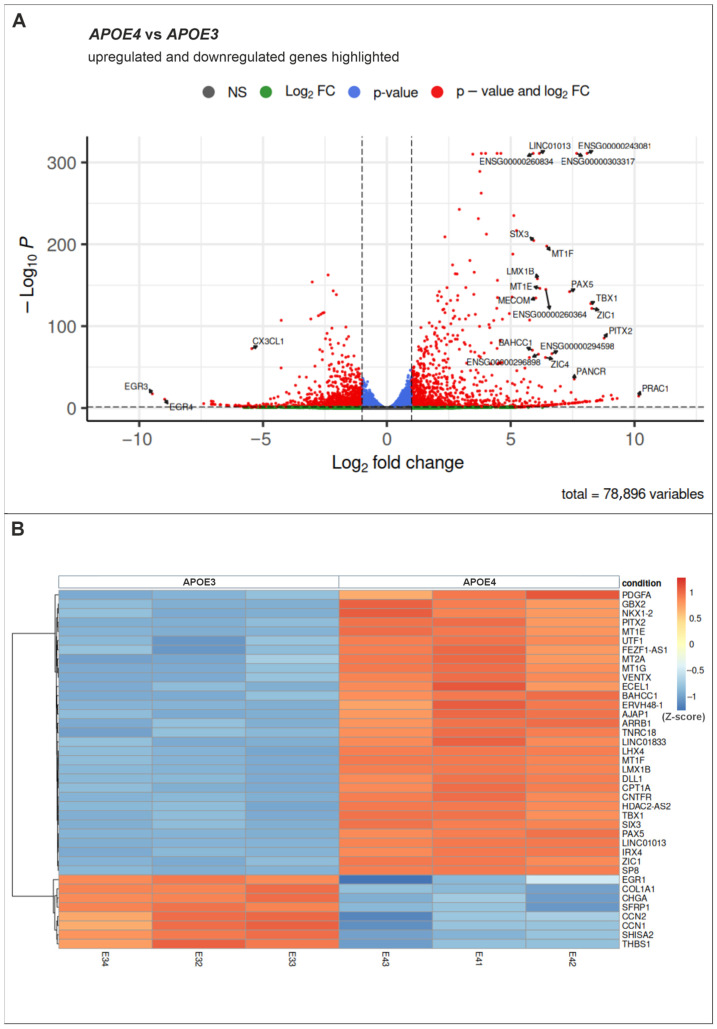
Differential gene expression analysis in *APOE4* cells vs. *APOE3* cells. (**A**) Volcano plot shows that 1105 genes are upregulated and 1324 genes are downregulated in *APOE4* cells compared to *APOE3* cells. *p*-value ≤ 0.05 and log2 fold change ≥ 1.0 (for upregulated genes) or ≤−1.0 (for downregulated genes). Dashed lines indicate these thresholds. (**B**) Heatmap of differentially expressed genes in *APOE3* and *APOE4* cells. Relative gene expression levels across samples. Data are Z-scores, where the color intensity represents the number of standard deviations a sample deviates from the mean expression of that specific gene. Each column represents a technical replicate (*n* = 3).

To further characterize transcriptional differences between *APOE3* and *APOE4* cells, differential gene expression analysis was performed, and the results are visualized using a volcano plot (Figure 2A). In total, 1105 genes were upregulated and 1324 genes were downregulated in *APOE4* cells compared to *APOE3* cells. These results indicate that the *APOE* allele has a significant impact on the global gene expression profile of induced pluripotent cells. A heatmap of selected differentially expressed genes further highlights clear transcriptional differences between *APOE3* and *APOE4* cells (Figure 2B). Notably, several genes associated with neural cell fate determination are upregulated in *APOE4* cells, including *ZIC1* (zinc finger protein of the cerebellum 1), *GBX2* (gastrulation brain homeobox 2), and *SIX3* (SIX homeobox 3) [30,31,32]. Additionally, zinc-inducible metallothionein genes, which mediate the cellular stress response to reactive oxygen species (ROS), are significantly upregulated, including *MT1E* (metallothionein 1E), *MT2A* (metallothionein 2A), *MT1G* (metallothionein 1G), and *MT1F* (metallothionein 1F) [33]. A detailed list of the top 200 up- and downregulated genes is provided in the Supplementary Materials (Tables S1 and S2).

### 2.2. Developmental Pathways Are Preferentially Enriched in APOE4 Cells

**Figure 3:**
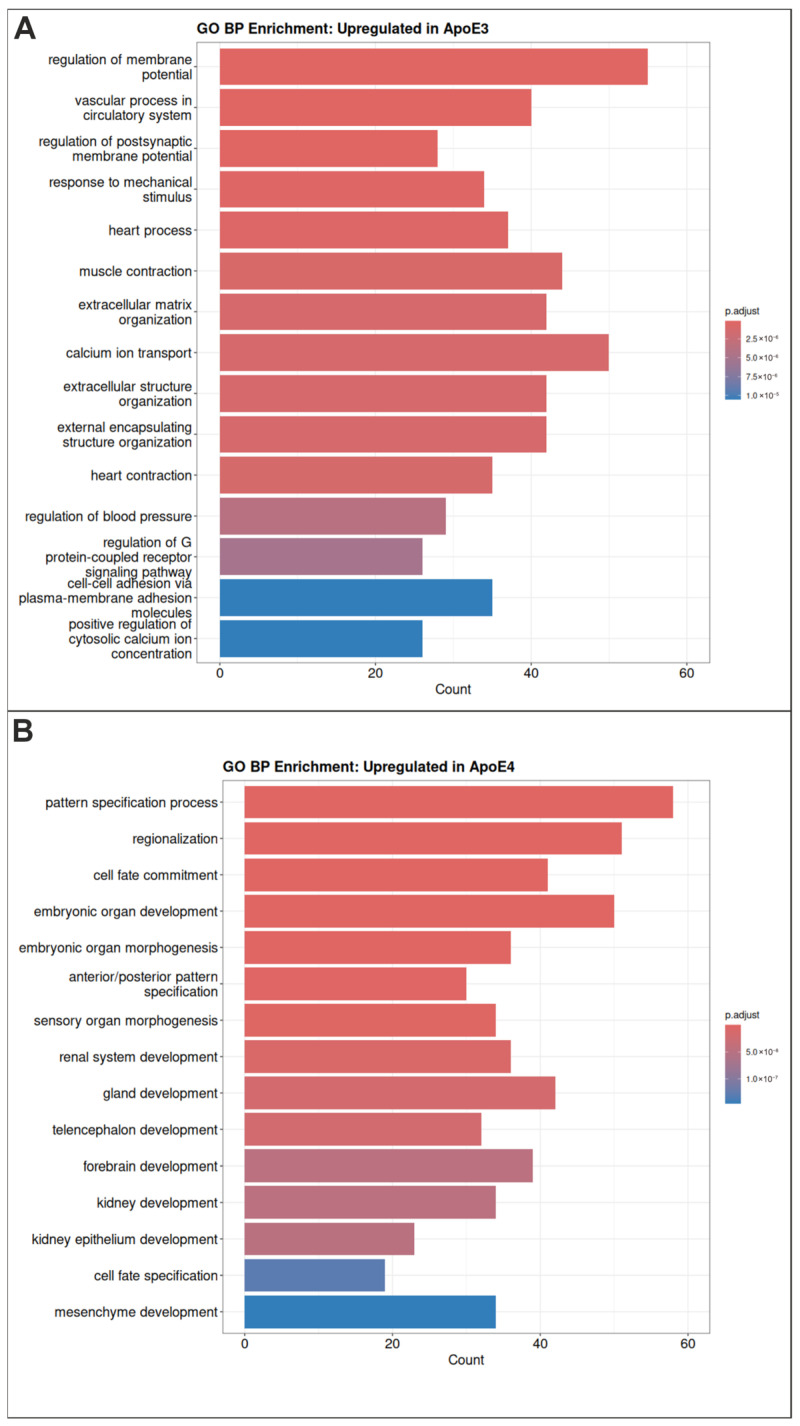
GO term enrichment regarding biological processes. (**A**) Upregulated genes in *APOE3* cells are enriched for membrane-associated, muscle-related, and ECM processes. (**B**) Upregulated genes in *APOE4* cells are enriched for developmental processes.

Gene Ontology (GO) enrichment analysis of the upregulated genes revealed distinct biological signatures in *APOE3* and *APOE4* cells (Figure 3). While genes upregulated in *APOE3* cells are enriched for cardiovascular and contractile functions, calcium-dependent signaling and regulation of membrane potential, as well as extracellular matrix (ECM) organization and cell adhesion (Figure 3A), genes upregulated in *APOE4* cells are predominantly associated with developmental and differentiation processes (Figure 3B).

**Figure 4:**
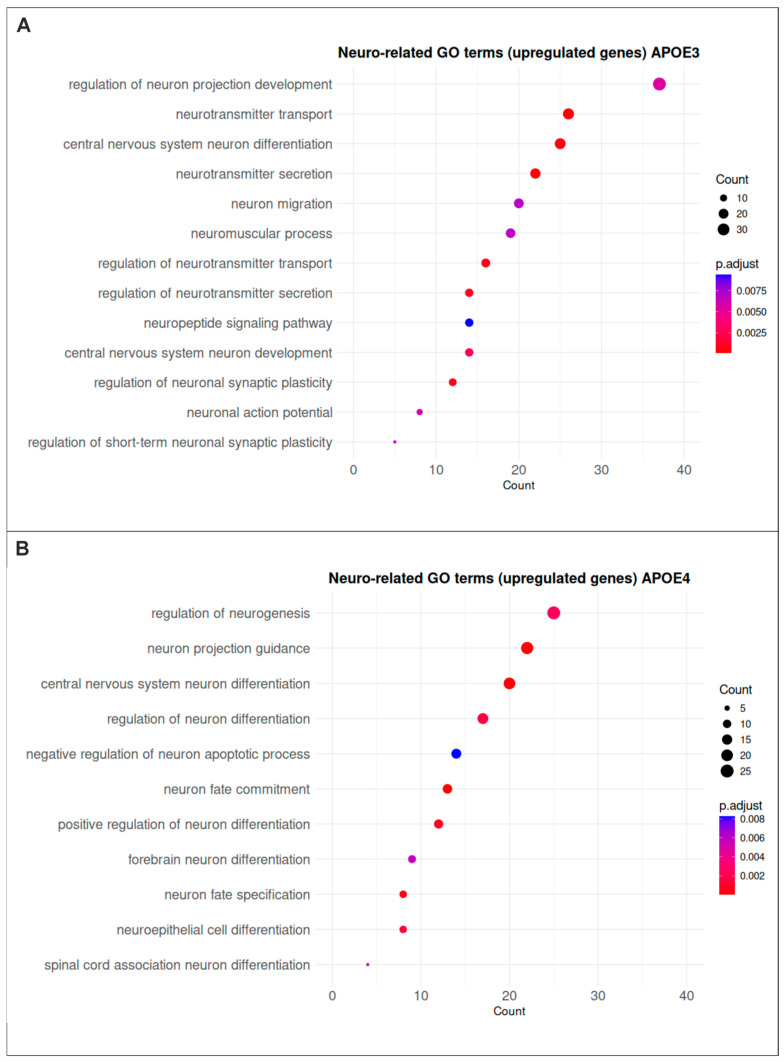
GO term enrichment regarding neuro-related processes in *APOE3* and *APOE4* cells. (**A**) Neuro-related GO terms enriched among gens upregulated in *APOE3* cells include processes associated with neuronal development and differentiation, synaptic signaling and neurotransmitter transport, as well as neuronal activity and synaptic plasticity. (**B**) In contrast, genes upregulated in *APOE4* cells are predominantly enriched for GO terms related to neuronal development and differentiation.

Given the neurodegenerative nature of AD, a focused analysis of neuro-related GO terms was performed. It revealed that genes upregulated in *APOE3* cells are enriched for processes related to neuronal development and differentiation, as well as synaptic signaling, neurotransmitter transport, neuronal activity, and synaptic plasticity (Figure 4A). In contrast, genes upregulated in *APOE4* cells are predominantly associated with neuronal developmental processes (Figure 4B).

### 2.3. Protein Interaction Networks Reveal Neurodevelopmental and Metallothionein Gene Clusters

**Figure 5:**
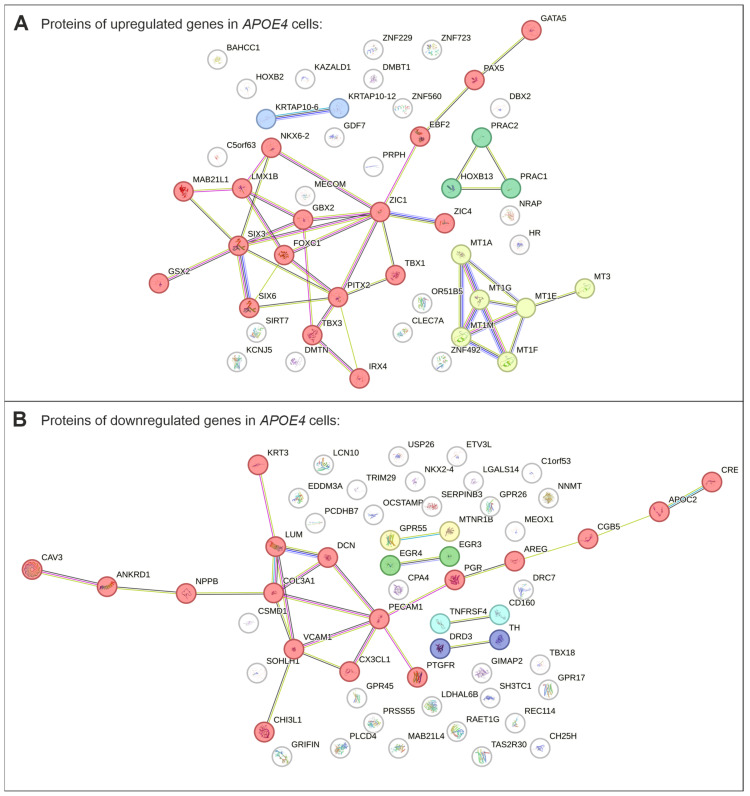
Protein network analysis based on the upregulated and downregulated genes in *APOE4* cells compared to *APOE3* cells. (**A**) The protein network of upregulated genes in *APOE4* cells reveals two major clusters: a large cluster enriched for genes involved in neurodevelopmental processes (red) and a smaller cluster consisting of metallothioneins (yellow). (**B**) The protein network of downregulated genes reveals a large cluster containing genes associated with extracellular matrix organization (red).

Protein–protein interaction network analysis of the top 200 up- and downregulated genes was performed to investigate functional relationships among the differentially expressed genes (Figure 5). The resulting network revealed two major clusters among the upregulated genes in *APOE4* cells (Figure 5A).

A large cluster comprised proteins associated with neurodevelopmental processes and neuronal differentiation (in red), consistent with the enrichment of neural Gene Ontology terms observed in the transcriptomic analysis (Table 1).

In addition, a smaller cluster consisting of metallothionein family members was identified, including MT1A, MT1G, MT1M, MT1F, MT1E, MT3 (in yellow). Metallothioneins are known to play important roles in cellular responses to metal ions and oxidative stress [33]. These results suggest that the transcriptional changes observed in *APOE4* cells involve coordinated regulation of genes associated with neuronal lineage specification as well as cellular stress response. A similar analysis was performed for the downregulated genes to investigate potential functional relationships within this group (Figure 5B). The resulting network revealed a large cluster of proteins associated with extracellular matrix organization and cell adhesion (in red), consistent with the GO enrichment analysis indicating reduced expression of genes involved in structural and contractile processes in *APOE4* cells (Table 2).

### 2.4. Neural Induction of APOE3 and APOE4 Cells

**Figure 6:**
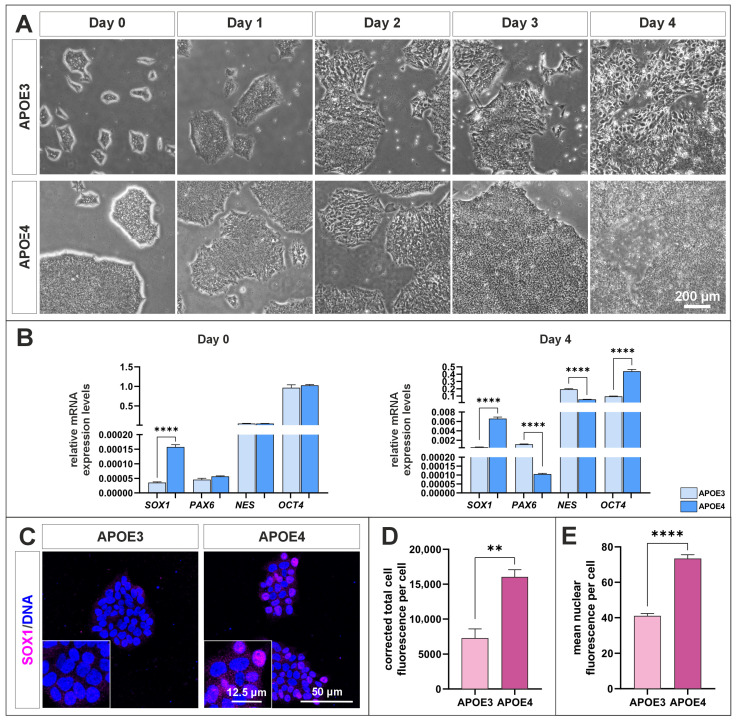
Analysis of differences in neural induction of *APOE3* and *APOE4* cells. (**A**) Morphological changes in *APOE* cells after neural induction for four days. (**B**) Early neural marker expression in *APOE* cells before and after neural induction (**C**) Immunocytochemical staining of SOX1 in *APOE3* and *APOE4* cells. (**D**) Quantification of fluorescence intensity demonstrated significantly increased SOX1 expression in *APOE4* cells compared to *APOE3* cells. (**E**) Nuclear SOX1 signal was also significantly elevated in *APOE4* cells relative to *APOE3* cells. Statistical analysis was performed using the Welch’s *t*-test (*n* = 3). **** *p* ≤ 0.0001, ** *p* ≤ 0.01. Data are presented as mean ± standard error of the mean.

Induction of neural differentiation in *APOE3* and *APOE4* cells over four days resulted in observable morphological changes in both cell lines by day 2 post-induction (Figure 6A). While *APOE3* cells continued to undergo morphological transformation until day 4, *APOE4* cells exhibited increased proliferation and a partial reversion toward their original morphology. Gene expression analysis of early neural markers, including SRY-box transcription factor 1 (*SOX1*) [34], Paired box gene (*PAX6*) [35], and Nestin (*NES*), revealed that prior to induction, *SOX1* expression was significantly upregulated in *APOE4* cells compared to *APOE3* cells (Figure 6B). After four days of neural induction, *PAX6* and *NES* expression levels were significantly higher in *APOE3* cells relative to *APOE4* cells. Although the pluripotency marker decreased in both cell lines, its expression remained significantly lower in *APOE3* cells compared to *APOE4* cells. Notably, *SOX1* expression remained higher in *APOE4* cells than in *APOE3* cells even after neural induction. Immunocytochemical analysis of SOX1 expression revealed a noticeably stronger fluorescence signal in *APOE4* cells compared to *APOE3* cells (Figure 6C). SOX1 is among the earliest markers of neuroectodermal differentiation. In *APOE4* cells, fluorescence intensity appeared increased both in the cytoplasm (Figure 6D) and in the nucleus (Figure 6E), whereas *APOE3* cells showed overall weaker staining. These results indicate increased SOX1 protein abundance in *APOE4* cells.

### 2.5. APOE4 Cells Exhibit Altered Regulation to EGR Genes

**Figure 7:**
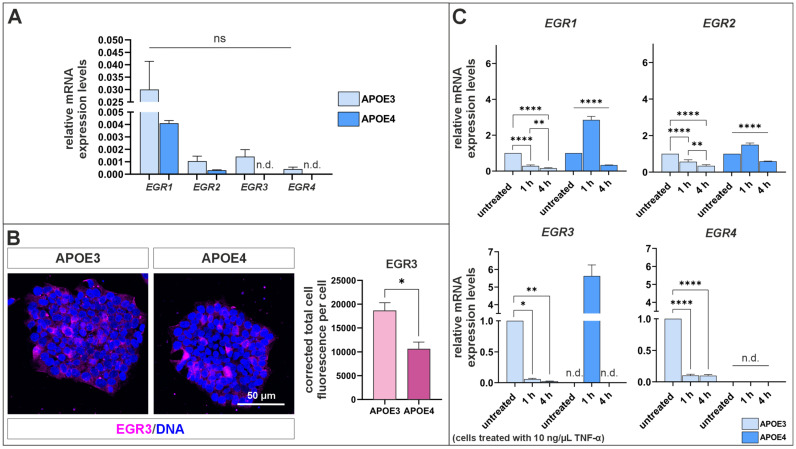
The *APOE4* genotype affects the expression of *EGR* family genes in iPSCs. (**A**) All four genes of the *EGR* family are downregulated in untreated *APOE4* cells compared to *APOE3* cells. (**B**) At protein level, significantly lower levels of EGR3 are observed in *APOE4* cells compared to *APOE3* cells. (**C**) Following treatment with TNF-α, the expression of all *EGR* genes is significantly downregulated in *APOE3* cells. In *APOE4* cells, the expression of *EGR1-EGR3* initially increases after 1 h of TNF-α treatment and subsequently decreases below baseline levels after 4 h. Genes with Ct values > 37 were considered not expressed (n.d. = not detectable; *EGR3* and *EGR4* in *APOE4* cells). Statistical analysis of qPCR data was performed using one-way ANOVA (*n* = 6), followed by multiple comparisons corrected using the False Discovery Rate (FDR) method of Benjamini, Krieger, and Yekutieli. ns = not significant; * *q* ≤ 0.05, ** *q* ≤ 0.01, **** *q* ≤ 0.0001. Statistical analysis of immunocytochemistry was performed using the Welch’s *t*-test test (*n* = 3). * *p* ≤ 0.05. Data are presented as mean ± standard error of the mean.

Interestingly, several members of the early growth response (*EGR1-EGR4*) family were found to be downregulated in *APOE4* cells in the RNA-seq dataset. Based on this observation, the expression of *EGR* genes was investigated in more detail (Figure 7). Consistent with the RNA-seq data, RT-qPCR analysis confirmed that the expression of *EGR* genes is downregulated in *APOE4* cells compared to *APOE3* cells (Figure 7A). In contrast, the expression of *EGR3* and *EGR4* could not be reliably detected in either condition (Ct value > 37). At the protein level, a significantly reduced expression of *EGR3* was also observed in *APOE4* cells (Figure 7B). In addition, the expression of *EGR* genes was analyzed 1 h and 4 h after TNF-α stimulation (Figure 7C). While the expression of all *EGR* genes decreased in *APOE3* cells upon TNF-α stimulation, the expression of *EGR1-EGR3* was significantly increased in *APOE4* cells after 1 h of treatment before declining again after 4 h. *EGR4* expression was not detectable in *APOE4* cells at any time point during TNF-α treatment.

### 2.6. APOE Genotype Influences the TNFR1/TNFR2 Expression Balances

**Figure 8:**
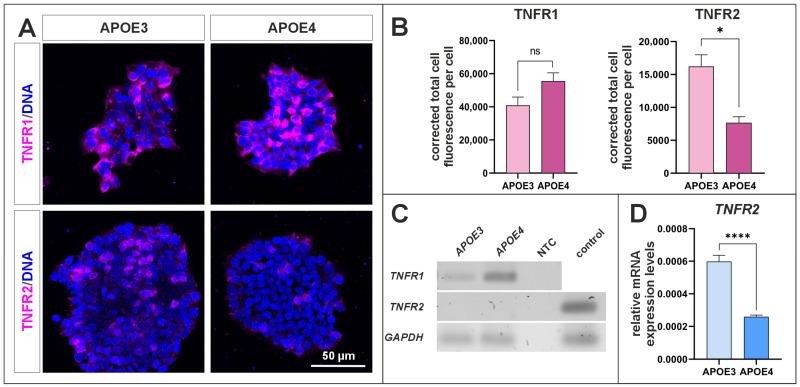
TNFR1 and TNFR2 expression analysis in *APOE3* and *APOE4* cells. (**A**) Immunocytochemical staining of TNFR1 and TNFR2 in APOE3 and APOE4 cells indicates a visually higher expression of TNFR1 in APOE4 cells, accompanied by a lower expression of TNFR2 compared to APOE3 cells. (**B**) Quantification of the ICC images confirms a significantly higher expression of TNFR2 in APOE3 cells. (**C**) Agarose gel electrophoresis of amplified cDNA shows expression of *TNFR1* in both cell lines, whereas no expression of *TNFR2* was detected. NTC indicates the no-template control; the control sample represents a positive control derived from inferior turbinate stem cells (ITSCs). (**D**) On mRNA level *TNFR2* is significantly higher expressed in *APOE3* cells compared to *APOE4* cells. Statistical analysis was performed using the Welch’s *t*-test test (*n* = 3). * *p* ≤ 0.05, **** *p* ≤ 0.0001. Data are presented as mean ± standard error of the mean.

Since TNF-α stimulation resulted in differential regulation of *EGR* gene expression between *APOE3* and *APOE4* cells, the expression of TNF receptors was investigated to determine whether differences in receptor abundance could contribute to the observed transcriptional responses (Figure 8). Immunocytochemical staining of TNFR1 and TNFR2 revealed a visually higher expression of TNFR1 compared to TNFR2 in both cell lines, whereas TNFR2 expression appeared reduced in *APOE4* cells compared to *APOE3* cells (Figure 8A). Quantification of the immunocytochemistry images confirmed a significantly higher expression of TNFR2 in *APOE3* cells (Figure 8B). Analysis of amplified cDNA by agarose gel electrophoresis detected expression of *TNFR1* in both lines, while *TNFR2* expression could not be detected under the analyzed conditions (Figure 8C). Nevertheless, analysis of mRNA levels revealed a significant higher expression of *TNFR2* in *APOE3* cells compared to *APOE4* cells (Figure 8D).

## 3. Discussion

In this study, we investigated the transcriptomic landscape of pluripotent stem cells carrying either the *APOE3* or the *APOE4* genotype. The *APOE4* allele represents the strongest genetic risk factor for late-onset AD and is associated with a markedly increased probability of developing the disease in aging individuals when present in the homozygous state [20]. Our results demonstrate that the *APOE4* genotype alters gene expression patterns already in pluripotent cells, suggesting a potential influence of *APOE4* on early developmental processes. Human *APOE4* carriers exhibit measurable alterations in brain structure and function as early as infancy, with regional effects that may persist across development. In infants and toddlers, *APOE4* has been associated with reduced gray matter volume and lower myelin water fraction in brain regions that are vulnerable to AD [36,37]. Children, homozygous for *APOE4*, show poorer performance in executive function-specifically cognitive flexibility and working memory, as assessed by the NIH Toolbox Cognition Battery-up to 8 years of age, but higher scores between 8 and 14 years, with no differences thereafter [38]. However, to the best of our knowledge, the impact of *APOE4* on human embryonic neurogenesis has not yet been examined in depth at the gene-expression level.

PCA after RNA sequencing revealed that PC1 accounted for the vast majority of variance (Figure 1), indicating a strong separation between *APOE3* and *APOE4* cells. That suggests that the APOE genotype represents a major determinant of the observed transcriptional differences. Nevertheless, additional factors such as cell cycle state, clonal variability, or stochastic gene expression may also contribute. Increased dispersion of *APOE4* samples along PC2 further indicates greater transcriptional heterogeneity.

Notably, enrichment analysis revealed a significant downregulation of synaptic-related pathways in *APOE4* cells (Figure 3), despite the pluripotent state of the analyzed cells. While synaptic function is not yet established at this stage, these findings suggest that APOE4-associated transcriptional alterations may already impact gene networks required for later synaptic development. In this context, the impaired neurodevelopmental phenotype observed in *APOE4* cells may reflect an early molecular priming that predisposes cells to synaptic dysfunction at more advanced stages of differentiation.

Furthermore, an upregulation of genes associated with neural development as well as metallothionein family members in *APOE4* cells compared to *APOE3* cells were observed, whereas genes involved in ECM organization and cell adhesion were downregulated in *APOE4* cells. To further explore the functional relationships between the differentially expressed genes, a protein–protein interaction network analysis was performed using STRING (Figure 5). The network generated from the upregulated genes in *APOE4* revealed two clusters. The large cluster contained several proteins associated with neural development, including genes involved in early patterning and neuronal differentiation (Table 1). Proteins included in this cluster compromise, for example, zinc finger proteins of the cerebellum (ZIC) 1 and 4. In mice, ZIC1 has been shown to be required for normal cerebellar development. Furthermore, ZIC1 and ZIC4 have been reported to cooperate in the proliferation of the external granule layer (EGL) and in folial patterning of the cerebellum [39]. In humans heterozygous loss of *ZIC1* and *ZIC4* has been associated with Dandy-Walker malformation, a rare congenital anomaly affecting the cerebellum and posterior cranial fossa [39]. Member of the sine oculis homeobox (SIX) protein family are transcription factors involved in the development of multiple organs, including the head, retina, ear, nose, brain, kidney, muscle, and gonads. SIX3, in particular, plays a critical role in eye and forebrain development [40]. Mutations in *SIX3* have been linked to holoprosencephaly, a developmental disorder characterized by the failure of the forebrain to properly divide into bilateral cerebral hemispheres [41]. In addition, Genomic Screen Homeobox 2 (GSX2) is a homeodomain transcription factor that regulates developmental gene networks during embryogenesis. It plays an important role in dorsoventral patterning and neural fate specification. In human patients, two *GSX2* variants have been identified that are associated with severe intellectual disability, spastic tetraparesis, and dystonia [42]. Finally, Gastrulation Brain Homeobox 2 (GBX2) is transiently expressed in neurons during development and is essential for axon outgrowth, axon guidance, and the suppression of habenular neuronal identity [43].

The enrichment of these genes suggest that transcriptional programs related to neural lineage specification may already be activated in *APOE4* cells. This observation is consistent with the RNA-seq data and the increased expression and nuclear localization of SOX1 (Figure 6C–E), which represents an early marker of neuroectodermal commitment [44,45]. Recently; SOX1 was identified as a novel marker for peripheral neural stem cells (pNSCs) in mouse lungs [46]. These pNSCs, which are distinct from central nervous system NSCs, are specifically marked by SOX1 and co-expressed SOX2. The cells were found to distribute along the bronchi in both postnatal and adult mouse lungs, challenging the previous dogma that neural stem cells are restricted to the brain and spinal cord. These findings indicate differential neural differentiation capacities between *APOE3* and *APOE4* cells. While both genotypes initiate early morphological changes upon induction (Figure 6A), *APOE3* cells display a more sustained progression toward a neural phenotype, as reflected by continued morphological transformation and increased expression of *PAX6* and *NES* (Figure 6B). In contrast, *APOE4* cells exhibit elevated proliferation and partial reversion to their original morphology, suggesting an impaired or less stable commitment to neural differentiation. The persistently higher *SOX1* expression in *APOE4* cells, both prior to and following induction, may indicate a retention in an early neural progenitor-like state rather than progression toward more differentiated neural lineages. Additionally, the comparatively higher levels of the pluripotency marker in *APOE4* cells after induction further support the notion of incomplete differentiation. Together, these results suggest that the *APOE4* genotype may be associated with altered or delayed neural differentiation dynamics, potentially reflecting reduced differentiation efficiency or stability. Previous studies have shown that neurons differentiated from iPSCs derived from patients with sporadic AD exhibit accelerated differentiation accompanied by reduced renewal of progenitor cells [28]. In the same study, impaired nuclear translocation of the transcriptional repressor REST as well as alterations of the nuclear lamina were reported. In line with these findings, we previously demonstrated that the nuclear translocation of the transcription factor NF-κB is also impaired in *APOE4* cells [29]. Together, these observations suggest that APOE4-associated alterations may not only affect neuronal gene regulatory networks but may also contribute to dysregulation of inflammatory signaling pathways.

In addition to this developmental cluster, a second smaller cluster was composed primarily of metallothioneins. Metallothioneins are cysteine-rich proteins that play important roles in cellular heavy metal detoxification and redox homeostasis. Apart from binding metal ions, metallothioneins also act as potent antioxidants by scavenging free radicals and thereby reducing cellular stress caused by ROS [33]. Elevated ROS levels, indicative for oxidative stress, have previously been observed in neurons differentiated from iPSCs derived from AD patients [47]. A hallmark of AD is the accumulation of Aβ plaques, which contain high concentrations of metal ions such as copper and zinc. These metal ions can be bound by metallothioneins, suggesting a potential neuroprotective role of these proteins in the context of amyloid pathology [33]. Increased metallothionein expression is therefore often considered a marker of oxidative stress. Notably, elevated ROS levels have been reported to occur prior to extracellular Aβ deposition and to increase with age [47]. The upregulation of metallothionein genes observed in our dataset suggests that oxidative stress-related pathways may already be activated in pluripotent cells carrying the *APOE4* genotype, indicating that early cellular alterations associated with AD risk may occur at developmental stages preceding neuronal differentiation. However, while oxidative stress-related pathways were enriched at the transcriptomic level, direct assessment of intracellular ROS levels would be required to determine whether these changes translate into a functional redox imbalance.

In contrast, the protein–protein interaction network derived from downregulated genes in *APOE4* cells was dominated by a large cluster containing proteins involved in extracellular matrix organization, cell adhesion and cytoskeletal regulation (Table 2). These processes are essential for maintaining cell–cell interactions and structural integrity of the cellular microenvironment. Reduced expression of ECM-related genes may therefore indicate alterations in cell adhesion and microenvironmental signaling [48]. For example, *COL3A1*, encoding the collagen alpha-1(III) chain, forms type III collagen as a homotrimer. Type III collagen accounts for approximately 5–20% of total collagen in the human body and contributes to the structural stability of various organs and connective tissue [49]. Next, decorin (*DCN*; belongs to the class of small leucine-rich proteoglycans (SLRPs) within the ECM. Decorin is expressed in cartilage tissue and represents an important component of the ECM. Silencing of *DCN* in human chondrocytes has been shown to affect cell adhesion, cell growth, and ECM metabolism, while also inhibiting cell proliferation, adhesion and inducing apoptosis [50]. Furthermore, *LUM* was found to be downregulated in *APOE4* cells. Lumican (*LUM*) is a structural and functional component of the ECM a class II SLRP. Studies in *LUM* knockout mice demonstrated disrupted ECM organization and a loss of optical properties of the cornea, highlighting its importance for tissue integrity [51]. Finally, vascular cell adhesion molecule-1 (VCAM1), a member of the immunoglobulin superfamily, functions as an important cell adhesion molecule and is expressed on the surface of activated endothelial cells [52]. In the context of AD, VECAM-1 has been proposed as a potential biomarker, as its plasma levels have been reported to be elevated in patients with AD compared with individuals with mild cognitive impairment [53]. These findings suggest that the *APOE* genotype may influence transcriptional programs that are already active at early developmental stages.

Next, we analyzed the expression of the *EGR* gene family across the different cell lines (Figure 7). *EGR1-EGR4* were downregulated in *APOE4* cells compared to *APOE3* cells (Figure 7A), and EGR3 protein levels were significantly reduced in *APOE4* cells (Figure 7B). EGRs belong to the immediate early genes (IEGs), which are rapidly induced upon stimulation and regulate transcriptional programs through three zinc-finger motifs within their DNA-binding domain [54,55]. They are involved in fundamental cellular processes, including cell growth, differentiation, apoptosis, and stress responses [56], as well as neuronal functions such as synaptic plasticity, learning and memory formation [55]. In mice, *EGR1* knockout results in impairments in spatial learning and spatial recognition memory [54,57]. Similarly, reduced hippocampal *EGR1* expression in rats disrupts memory reconsolidation but not consolidation [58,59]. Interestingly, in AD mouse models, inhibition of *EGR1* has been shown to reduce AD pathology and improve cognitive function [55]. In contrast, *EGR2* plays a key role in myelination in the peripheral nervous system. *EGR2* knockout mouse embryos display impaired hindbrain segmentation, and EGR2 is activated in Schwann cells prior to the onset of myelination; its loss results in defective peripheral myelination [60,61]. EGR3 is considered a major regulator of biological processes related to memory and synaptic plasticity. It regulates other members of the *EGR* family, including *EGR1*, *EGR2*, *EGR4*, and *NAB2*, through a feedback loop. Dysregulation of this network has been associated with impairments in memory (particularly short-term memory [62]), synaptic plasticity, immune function, growth factor-mediated processes, myelination and vascularization [63]. The functional role of *EGR4* in neuronal processes remains less well characterized, although EGR family members are activity-induced transcription factors that contribute to synaptic plasticity-related gene regulation.

The pro-inflammatory cytokine TNF-α is known to activate transcriptional programs through pathways such as MAPK and NF-κB, which frequently induce IEGs including members of the *EGR* family [56,64]. In our study, TNF-α treatment resulted in reduced *EGR* expression in pluripotent *APOE3* cells (Figure 7C), whereas *EGR1*, *EGR2* and *EGR3* were significantly induced in *APOE4* cells after one hour of stimulation and decreased again after four hours of stimulation. The four-hour time point was selected to capture the later phase of NF-κB signaling, which reflects sustained signaling and downstream transcriptional responses beyond the initial activation observed at earlier time points. While early responses appear attenuated, the induction of EGRs at later time points suggests that transcriptional activation is not abolished but rather delayed. This further implies, that feedback mechanisms remain functionally intact. Altered *EGR1* expression has previously been reported in AD, suggesting that dysregulation of EGR-dependent transcription may contribute to AD-related transcriptional changes [55,65]. Moreover, impaired EGR3 signaling has been associated with neuronal dysfunction and cognitive deficits [66,67]. Together, these findings support the idea that inflammatory signaling, including TNF-α pathways, may differentially regulate EGR-dependent transcriptional programs and thereby contribute to altered cellular differentiation.

Furthermore, we investigated the expression of the TNF-α receptors TNFR1 and TNFR2 (Figure 8). TNFR1 is known to mediate pro-inflammatory and apoptotic signaling pathways and has been associated with neurodegenerative processes [68]. In our analysis, however, TNFR1 expression did not differ significantly between *APOE3* and *APOE4* cells. (Figure 8A,B). In contrast, TNFR2 protein levels were significantly reduced in *APOE4* cells. TNFR2 signaling has been linked to neuroprotective effects, including the promotion of neuronal survival, modulation of neuroinflammation, and support of remyelination processes [68,69]. The reduced TNFR2 expression observed in *APOE4* cells may therefore indicate a shift in TNF signaling towards a more pro-inflammatory state. Alterations in the balance between TNFR1- and TNFR2-mediated signaling have been proposed to contribute to neuroinflammatory mechanisms involved in neurodegenerative diseases such as AD [69].

**Figure 9:**
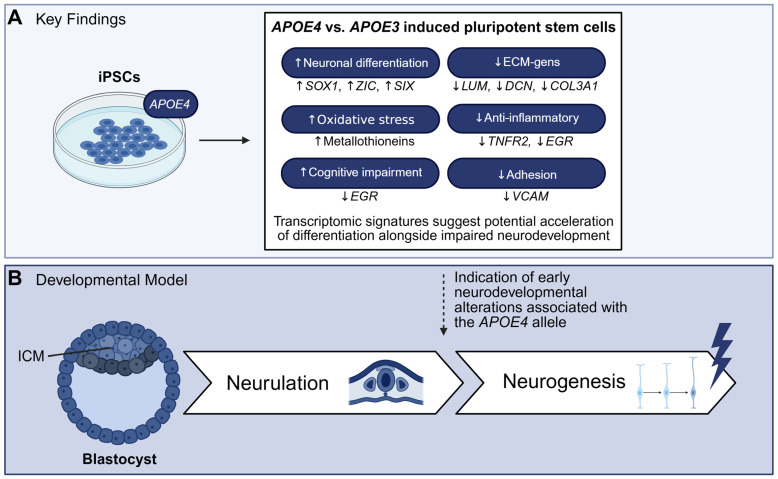
Summary of the transcriptional changes in induced pluripotent stem cells homozygous for *APOE4*. (**A**) Schematic overview of the key findings of this study. *APOE4* cells exhibit increased expression of genes associated with neural development and stress responses, accompanied by reduced expression of ECM and cell-adhesion-related genes. Increased *SOX1* expression suggest early neural lineage priming. In addition, altered *EGR* gene expression and reduced *TNFR2* levels indicate dysregulated stress and inflammatory signaling. (**B**) Depiction of early human development from the blastocyst stage, highlighting the inner cell mass (ICM), through neurulation to neurogenesis. The *APOE4* genotype may affect early neurodevelopmental processes, potentially through altered transcriptional regulation and signaling pathways identified in this study.

Taken together, the results of this study suggest the *APOE* genotype influences transcriptional programs already at the pluripotent stage (Figure 9). Although the use of isogenic lines minimizes genetic variability, we cannot fully exclude contributions from epigenetic or cell state differences. Nevertheless, transcriptomic analyses revealed an enrichment of genes associated with neural development as well as metallothioneins in *APOE4* cells compared to *APOE3* cells, indicating potential alterations in early differentiation programs and cellular stress responses. Consistent with these findings, increased expression and nuclear localization of SOX1 further suggest a shift towards early neural lineage commitment. However, the concurrent activation of stress-related pathways, including altered expression of *EGR* transcription factors and reduced TNFR2 levels, points towards dysregulated signaling responses to inflammatory and oxidative stimuli. The upregulation of metallothionein genes in *APOE4* cells further suggest increased oxidative stress, which has been implicated in the modulation of cellular differentiation processes. Collectively, these observations suggest that *APOE4* cells may exhibit premature neural lineage priming in response to elevated cellular stress, which could result in the activation of aberrant differentiation pathway.

## 4. Materials and Methods

### 4.1. Cell Culture

All experiments were conducted using the homozygous iPS cell line JIPSC001150 and its genetically corrected counterpart JIPSC001162. Both sourced from The Jackson Laboratory (Bar Harbor, ME, USA). Cells were expanded on vitronectin-coated (5 µg/mL; Thermo Fisher Scientific, Waltham, MA, USA) 6-well culture plates (Sarstedt, Nürmbrecht, Germany). And maintained in E8 Flex medium (Thermo Fisher Scientific, Waltham, MA, USA) under standardized culture conditions. Cultures were kept at 37 °C in a humidified atmosphere containing 5% CO_2_ (Binder, Tuttlingen, Germany). Routine passaging followed the recommended protocol provided by the medium supplier. Pluripotency was verified through morphological assessment and analysis of established pluripotency markers.

### 4.2. RNA Sequencing

The iPSC cell lines were seeded in triplicate into vitronectin-coated 6-well plates and cultured until reaching a confluency of 70–90%. Cells were subsequently detached using a cell scraper and total RNA was isolated using NucleoSpin RNA Kit (Macherey-Nagel GmbH & Co. KG, Düren, Germany) according to the manufacturer’s instructions. Library preparation and sequencing were performed by Novogene (Cambridge, UK). Paired-end clean reads were aligned to the human reference genome (hg38) using HISAT2 (v2.2.1). Read counts for genomic features were obtained using FeatureCounts (v2.0.0). Differential gene expression analysis between experimental conditions was performed using DESeq2 package in R (v4.5.2). EnhancedVolcano (v3.17) was used to generate volcano plots, and ggplot2 in R was used for principal component analysis (PCA) visualization. Gene Ontology enrichment analysis was conducted using enrichGO package with the org.Hs.eg.db annotation database in R. Heatmap analysis was performed using the pheatmap package in R. Protein–protein-interaction network was generated by STRING (www.string-db.org; v12.0) and information on protein function was obtained from UniProt database (www.uniprot.org).

### 4.3. TNF-α Treatments

For TNF-α stimulation assays, cells were seeded onto µ-Slide 8-well chambers (Ibidi, Gräfelfing, Germany) and cultured for 48 h under standard conditions. Prior to stimulation, the culture medium was aspirated, cells were washed once with 1× phosphate-buffered saline (PAN-Biotech, Aidenbach, Germany), and fresh medium containing TNF-α (10 ng/µL; PeproTech, Cranbury, NJ, USA) was added. Cells were treated for the indicated time points ranging from 10 min to 4 h. Following stimulation, cells were washed with PBS and processed for immunocytochemical analysis.

### 4.4. Neural Induction

Neural induction was performed by seeding cells into individual wells of a 6-well plate and culturing them in E8 Flex medium for two days until they reached approximately 40–50% confluency. Neural differentiation was then initiated using neural induction medium (NIM), consisting of KnockOut™ DMEM (Thermo Fisher Scientific, Waltham, MA, USA), KnockOut™ Serum Replacement (Thermo Fisher Scientific, Waltham, MA, USA), GlutaMAX™ (Thermo Fisher Scientific, Waltham, MA, USA), non-essential amino acids (Thermo Fisher Scientific, Waltham, MA, USA), and STEMdiff™ SMADi Neural Induction Supplement (STEMCELL Technologies, Vancouver, BC, Canada). After four days of induction, total RNA was isolated for subsequent qPCR analysis.

### 4.5. Immunocytochemistry

Cells were fixed in 4% paraformaldehyde for 10 min at room temperature, washed three times with 1× PBS, and blocked for 45 min in PBS containing 0.02% Triton X-100 (Sigma-Aldrich, Darmstadt, Germany) and 5% goat serum (Dianova, Hamburg, Germany). Primary antibodies were applied for 1 h at room temperature (Table 3), followed by PBS washes and incubation with Alexa Flour-conjugated secondary antibodies (Alexa Fluor 555 goat anti-rabbit and Alexa Fluor 555 donkey anti-goat; both Thermo Fisher Scientific, Waltham, MA, USA) at a dilution of 1:300. Samples were mounted using a DAPI-containing mounting medium (Ibidi, Gräfelfing, Germany) and imaged with a Leica STELLARIS 8 FALCON confocal laser scanning microscope (Leica Microsystems, Wetzlar, Germany). Image processing and statistical analysis were performed using ImageJ (version 1.54p; National Institutes of Health, Bethesda, MD, USA), CorelDRAW 2023 (Corel Corporation, Ottawa, ON, Canada), and GraphPad Prism 8.3.0 (GraphPad Software, Inc., San Diego, CA, USA).

### 4.6. RT-qPCR

Isolated RNA was transcribed into cDNA using the First Strand cDNA Synthesis Kit (Thermo Fisher Scientific, Waltham, MA, USA). For all qPCR reactions the PowerUp™ SYBR™ Green Master Mix (Thermo Fisher Scientific, Waltham, MA, USA) was used. For each reaction, triplicates were prepared in the LightCycler^®^ 480 System (Roche, Basel, Switzerland). The primer sequences can be found in Table 4 *GAPDH* and *RPLP0* served as housekeeping genes. Microsoft Excel 2019 (Microsoft, Redmond, WA, USA) and finally GraphPad Prism 8.3.0 were used for analysis.

## Figures and Tables

**Table 1:** Functional annotation of proteins from the major upregulated cluster in *APOE4* cells based on UniProt database entries.

Protein	Function
EBF2	Transcription factor that regulates osteoclast differentiation.
FOXC1	Transcription factor that plays a role cellular and developmental processes such as eye, bones, cardiovascular, kidney and skin development
GATA5	Transcription factor required during cardiovascular development
GBX2	Transcription factor for cell pluripotency and differentiation in the embryo
GSX2	Transcription factor that regulates the expression of numerous genes including genes important for brain development
IRX4	Important mediator of ventricular differentiation during cardiac development
LMX1B	Transcription factor involved in the regulation of podocyte-expressed genes
MAB21L1	Putative nucleotidyltransferase required for several aspects of embryonic development including normal development of the eye
NKX6-2	Transcription factor with repressor activity involved in the regulation of axon-glial interactions at myelin paranodes in oligodendrocytes
PAX5	Transcription factor that plays an essential role in commitment of lymphoid progenitors to the B-lymphocyte lineage
PITX2	play a role in myoblast differentiation
SIX3	Plays a role in eye development by suppressing WNT1 expression and in dorsal-ventral patterning by repressing BMP signaling pathway
SIX6	involved in eye development
TBX1	Transcription factor that plays a key role in cardiovascular development by promoting pharyngeal arch segmentation during embryonic development; involved in craniofacial muscle development
TBX3	Transcriptional repressor involved in developmental processes
ZIC1	Acts as a transcriptional activator. Involved in neurogenesis. Plays important roles in the early stage of organogenesis of the CNS, as well as during dorsal spinal cord development and maturation of the cerebellum
ZIC4	Transcription factor for central nervous system development

**Table 2:** Functional annotation of proteins from the major downregulated cluster in *APOE4* cells based on UniProt database entries.

Protein	Function
ANKRD1	May play an important role in endothelial cell activation. May act as a nuclear transcription factor that negatively regulates the expression of cardiac genes.
APOC2	Plays an important role in lipoprotein metabolism as an activator of lipoprotein lipase.
AREG	Ligand of the EGF receptor/EGFR. Autocrine growth factor as well as a mitogen for a broad range of target cells including astrocytes, Schwann cells and fibroblasts.
CAV3	May act as a scaffolding protein within caveolar membranes. Interacts directly with G-protein alpha subunits and can functionally regulate their activity.
CGB5	Has an essential role in pregnancy and maternal adaptation. Stimulates the ovaries to synthesize the steroids that are essential for the maintenance of pregnancy.
CHI3L1	Carbohydrate-binding lectin with a preference for chitin. Has no chitinase activity. May play a role in tissue remodeling and in the capacity of cells to respond to and cope with changes in their environment.
COL3A1	Collagen type III occurs in most soft connective tissues along with type I collagen. Involved in regulation of cortical development.
CREB	Phosphorylation-dependent transcription factor that is involved in different cellular processes including the synchronization of circadian rhythmicity and the differentiation of adipose cells
CX3CL1	Chemokine that acts as a ligand for both CX3CR1 and integrins. The CX3CR1-CX3CL1 signaling exerts distinct functions in different tissue compartments, such as immune response, inflammation, cell adhesion and chemotaxis
DCN	May affect the rate of fibrils formation
KRT3	Cytoskeletal and microfibrillar keratin
LUM	Collagen binding, extracellular matrix structural constituent
NPPB	Cardiac hormone that plays a key role in mediating cardio-renal homeostasis
PECAM1	Cell adhesion molecule which is required for leukocyte transendothelial migration (TEM) under most inflammatory conditions
PGR	The steroid hormones and their receptors are involved in the regulation of eukaryotic gene expression and affect cellular proliferation and differentiation in target tissues.
PTGFR	Receptor for prostaglandin F2-alpha (PGF2-alpha).
VCAM1	Cell adhesion glycoprotein predominantly expressed on the surface of endothelial cells that plays an important role in immune surveillance and inflammation

**Table 3:** Primary antibodies used for ICC in this study.

Primary Antibody	Host	Dilution	Manufacturer
SOX1	Goat	1:200	R&D Systems, Minneapolis, MN, USA
EGR3	Rabbit	1:100	Bioss, Woburn, MA, USA
TNFR1	Rabbit	1:50	Thermo Fisher Scientific, Waltham, MA, USA
TNFR2	Rabbit	1:200	Thermo Fisher Scientific, Waltham, MA, USA

**Table 4:** Primer used for RT-qPCR.

Primer	Sequence (5′ to 3′)
*GAPDH* fwd	CATGAGAAGTATGACAACAGCCT
*GAPDH* rev	AGTCCTTCCACGATACCAAAGT
*RPLP0* fwd	TGGTCATCCAGCAGGTGTTCGA
*RPLP0* rev	ACAGACACTGGCAACATTGCGG
*SOX1* fwd	GAGTGGAAGGTCATGTCCGAGG
*SOX1* rev	CCTTCTTGAGCAGCGTCTTGGT
*PAX6* fwd	TGGCTCACCAAGGCGAAATA
*PAX6* rev	CCGAGCAGTTGAGTCATTCAG
*NES* fwd	CGCACCTCAAGATGTCCCTC
*NES* rev	CAGCTTGGGGTCCTGAAAGC
*OCT4* fwd	CGAAAGAGAAAGCGAACCAG
*OCT4* rev	GCCGGTTACAGAACCACACT
*EGR1* fwd	AGAAGGACAAGAAAGCAGACAAAAGTGT
*EGR1* rev	GGGGACGGGTAGGAAGAGAG
*EGR2* fwd	CTTTGACCAGATGAACGGAGTG
*EGR2* rev	AGCAAAGCTGCTGGGATATG
*EGR3* fwd	GACTCGGTAGTCCATTACAATCAG
*EGR3* rev	AGTAGGTCACGGTCTTGTTGCC
*EGR4* fwd	CGCCTAGGTCTGTGCGCTGC
*EGR4* rev	CGGAAAACTCGCTAAGGTGGAG
*TNFR2* fwd	GAACCAGCCACAGGCACCA
*TNFR2* rev	ACGATGCAGGTGACATTGAC

## Data Availability

The original data presented in the study are openly available in GitLab at https://gitlab.com/fazel.mehran96/rna_seq_alzheimer (accessed on 20 March 2026).

## Supplementary Materials

The following supporting information can be downloaded at: https://www.mdpi.com/article/10.3390/ijms27104325/s1.

## Abbreviations

The following abbreviations are used in this manuscript:

Aβ	Amyloid-beta
AD	Alzheimer’s Disease
ADAM10	A Disintegrin And Metalloproteinase 10
APOE	Apolipoprotein E
APP	Amyloid Precursor Protein
BACE1	Beta-site Amyloid Precursor Protein Cleaving Enzyme 1
CCL2	C-C motif Chemokine Ligand 2
COL3A1	Collagen Alpha-1(III) Chain
CTCF	Corrected Total Cell Fluorescence
CXCL10	C-X-C Motif Chemokine Ligand 10
DAPI	4′,6-diamidino-2-phenylindole
DCN	Decorin
ECM	Extracellular Matrix
EGR	Early Growth Response
ESC	Embryonic Stem cells
GBX2	Gastrulation Brain Homeobox 2
GSX2	Genomic Screen Homeobox 2
IL-1β	Interleukin-1 beta
iPS	Induced Pluripotent Stem (cells)
LOAD	Late-Onset Alzheimer’s Disease
LUM	Lumican
MT	Metallothinein
NF-κB	Nuclear Factor kappa-light-chain-enhancer of activated B cells
PCA	Principal Component Analysis
PSEN1/2	Presenilin 1 and 2
REST	Repressor Element-1 Silencing Transcription Factor
ROS	Reactive Oxygen Species
SCNT	Somatic Cell Nuclear Transfer
SIX	Sine Oculis Homeobox
SLRP	Small leucine-rich proteoglycans
SOX1	SRY-box Transcription Factor 1
TNFα	Tumor Necrosis Factor alpha
TNFR	Tumor Necrosis Factor Receptor
VCAM1	Vascular Cell Adhesion Molecule-1
ZIC	Zinc Finger Protein if the Cerebellum
